# *Aegilops tauschii* Accessions with Geographically Diverse Origin Show Differences in Chromosome Organization and Polymorphism of Molecular Markers Linked to Leaf Rust and Powdery Mildew Resistance Genes

**DOI:** 10.3389/fpls.2017.01149

**Published:** 2017-06-28

**Authors:** Maciej Majka, Michał T. Kwiatek, Joanna Majka, Halina Wiśniewska

**Affiliations:** ^1^Cereal Genomics Team, Department of Genomics, Institute of Plant Genetics, Polish Academy of SciencesPoznań, Poland; ^2^Plant Molecular Physiology and Cytogenetics Team, Department of Environmental Stress Biology, Institute of Plant Genetics, Polish Academy of SciencesPoznań, Poland

**Keywords:** *Aegilops tauschii*, *in situ* hybridization, leaf rust, markers-assisted selection, polymorphism, powdery mildew

## Abstract

*Aegilops tauschii* (2n = 2x = 14) is a diploid wild species which is reported as a donor of the D-genome of cultivated bread wheat. The main goal of this study was to examine the differences and similarities in chromosomes organization among accessions of *Ae. tauschii* with geographically diversed origin, which is believed as a potential source of genes, especially determining resistance to fungal diseases (i.e., leaf rust and powdery mildew) for breeding of cereals. We established and compared the fluorescence *in situ* hybridization patterns of 21 accessions of *Ae. tauschii* using various repetitive sequences mainly from the BAC library of wheat cultivar Chinese Spring. Results obtained for *Ae. tauschii* chromosomes revealed many similarities between analyzed accessions, however, some hybridization patterns were specific for accessions, which become from cognate regions of the World. The most noticeable differences were observed for accessions from China which were characterized by presence of distinct signals of pTa-535 in the interstitial region of chromosome 3D, less intensity of pTa-86 signals in chromosome 2D, as well as lack of additional signals of pTa-86 in chromosomes 1D, 5D, or 6D. *Ae. tauschii* of Chinese origin appeared homogeneous and separate from landraces that originated in western Asia. *Ae. tauschii* chromosomes showed similar hybridization patterns to wheat D-genome chromosomes, but some differences were also observed among both species. What is more, we identified reciprocal translocation between short arm of chromosome 1D and long arm of chromosome 7D in accession with Iranian origin. High polymorphism between analyzed accessions and extensive allelic variation were revealed using molecular markers associated with resistance genes. Majority of the markers localized in chromosomes 1D and 2D showed the diversity of banding patterns between accessions. Obtained results imply, that there is a moderate or high level of polymorphism in the genome of *Ae*. *tauschii* determined by a geographical origin, which we proved by cytogenetic and molecular markers analysis. Therefore, selected accessions might constitute an accessible source of variation for improvement of Triticeae species like wheat and triticale.

## Introduction

*Aegilops tauschii* Coss. (2n = 2x = 14) is a diploid wild species which is reported as a donor of the D-genome of cultivated bread wheat – *Triticum aestivum* L. (Kihara, 1944; McFadden and Sears, 1946). What is more, D-genome is also a part of several tetraploid (i.e., *Ae*. *cylindrica* Host and *Ae*. *ventricosa* Tausch.) and hexaploid [i.e., *Ae*. *juvenalis* (Thell.) Eig and *Ae*. *vavilovii* (Zhuk.)] Chennav. species of the genus *Aegilops*.

Among *Aegilops* species, *Ae*. *tauschii* has the widest geographic distribution, from Turkey on the West to Afghanistan and central Asia in the East, and thus adapted to diversified environmental conditions (Ogbonnaya et al., 2005; Kalia et al., 2016). *Ae. tauschii* encompasses two subspecies – *Ae. tauschii* Coss. subsp. *strangulata* (Eig) Tzvel. and *Ae*. *tauschii* subsp. *Tauschii* (Kalia et al., 2016). Subspecies *strangulata* is distributed from Transcaucasian region (Armenia, Azerbaijan) to the southeastern Caspian Sea region in Iran, whereas subspecies *tauschii* is native to the southwestern Caspian Iran and Afghanistan (Kihara et al., 1965; Ogbonnaya et al., 2005; Wang et al., 2013; Kalia et al., 2016). According to previous reports, it is assumed that *Ae. tauschii* ssp. *strangulata* is the ancestor of the wheat D-genome (Wang et al., 2013).

Recently, progressive genetic erosion and increasing number of races of pathogens destructive for cereals were observed. Hence, there is need to find new sources of variation for breeding of cereals. *Ae*. *tauschii* is an excellent source of novel genes to various biotic and abiotic stresses (Valkoun et al., 1985; Gill et al., 1986; Cox et al., 1992; Assefa and Fehrmann, 2004). Close evolutionary relationship, a variety of genes and relatively easy crossability make this species especially interesting for improvement of cultivated Triticeae species like wheat and triticale (Ogbonnaya et al., 2005). Considering breeding of cereals, one of the main goals is to transfer genes determining resistance to fungal diseases like leaf rust and powdery mildew from wild relatives into cultivated varieties (Gill et al., 1983, 1986; Dhaliwal et al., 1991).

Leaf rust, caused by *Puccinia triticina* Eriks., occurs on leaves and is one of the most destructive diseases of wheat in the world, because of the ability of the pathogen to adapt to diverse climatic conditions (Kolmer, 1996; Chen et al., 2013). It occurs regularly in the regions with humid and warm environmental conditions like continental Europe with a temperate climate and leads up to 30% of yield losses (Roelfs et al., 1992). It was reported that genetic engineering of host resistance is both profitable and safe for environment approach to control leaf rust (Assefa and Fehrmann, 2000; Kalia et al., 2016). According to the literature, *Ae*. *tauschii* is a source of four seedling resistance genes [*Lr21* (1D), *Lr32* (3D), *Lr39* (2D), *Lr42* (1D)] and one determining adult plant resistance [*Lr22a* (2D)].

Powdery mildew, caused by *Blumeria graminis* f. sp. *tritici*, is second globally important leaf disease not only in wheat but also in triticale breeding, because populations of the pathogen are very dynamic and continually change their virulence structure. It is very common in Europe, parts of Asia, and the southeastern part of North America, with high rainfalls and maritime or semi-continental climate, where powdery mildew occurs annually and leads to significant yield losses (Wang et al., 2016). So far, the most feasible means of controlling the disease is by host resistance (Bennett, 1984; Wang et al., 2016). Over 70 powdery mildew resistance (*Pm*) alleles at 50 loci have been formally designated (*Pm1*–*Pm54*), whereas genes *Pm2* (5D), *Pm19* (7D), *Pm34* (5D), and *Pm35* (5D) were originated from *Ae. tauschii* (Cowger et al., 2012; Wang et al., 2016). What is more, *Ae*. *tauschii* might constitute also a valuable source of *Pm43* gene (Majka et al., 2016), which was previously mapped on chromosome 2D of this species by Jia et al. (2013).

So far, many efforts have been undertaken to characterize genetically diverse accessions of *Ae. tauschii* using SSR markers also in terms of disease resistance (Naghavi et al., 2009; Chhuneja et al., 2010). Despite a large number of analyzed accessions (13 and 85, respectively) and SSR markers (21 and 51, respectively) used in this study, none of the markers were assigned to resistance genes. Singh et al. (2012) evaluated 63 accessions of *Ae. tauschii* for marker-trait association using 35 SSR markers, and identified six SSR markers significantly associated with leaf rust resistance genes (*Xcfd19, Xgdm35, Xgwm261, Xgwm515, Xcfd4*, and *Xgwm645*). It was reported that using this markers the germplasm collections can be initially screened and characterized to identify candidate genetic stocks and genomic regions (Singh et al., 2012). What is more, some microsatellites markers associated with leaf rust resistance genes localized in D-genome were reported for bread wheat (Huang et al., 2003; Hiebert et al., 2007; Thomas et al., 2010).

Karyotype and C-banding patterns of *Ae*. *tauschii* chromosomes were reported by many authors (Chennaveeraiah, 1960; Gill and Kimber, 1974; Iordansky et al., 1978; Teoh and Hutchinson, 1983; Friebe et al., 1992). What is more, *Ae. tauschii* chromosomes were also examined using *in situ* hybridization with various probes, like pTa71 (18S-5.8S-26S rDNA), pTa794 (5S rDNA), pAs1 (*Afa* family) and pSc119.2 (Badaeva et al., 2002; Kwiatek et al., 2013; Molnar et al., 2014). However, in two of this studies only one accession of *Ae*. *tauschii* was taken under investigations. So far, only Friebe et al. (1992) and Badaeva et al. (2002) using C-banding technique revealed polymorphism between respectively 15 and 12 accessions of *Ae*. *tauschii* with a different origin. The number and distribution of repetitive sequences used in previous studies for fluorescent *in situ* hybridization were not sufficient to cover the whole karyotype of this species. What is more, the number and distribution of rDNA loci in the Triticeae species are reported to be a conservative feature (Badaeva et al., 1996). Because of the great agronomic importance of the D-genome in bread wheat, this genome was well-studied on the cytogenetic level using different probes especially repetitive sequences with oligonucleotides amongst them.

D-genome chromosomes of wheat cultivar Chinese Spring were studied in terms of distribution of synthetic oligonucleotides (2–3 bp repeats) (Cuadrado et al., 2000, 2008). Among 12 analyzed sequences only two allowed to obtain distinctive hybridization signals. (AAG)_5_ revealed signals in chromosomes 1D, 2D and 7D, (AGG)_5_ single and weak signals in chromosomes 2D, whereas two other sequences (AC)_8_ and (GCC)_5_ revealed dispersed signals scattered along the chromosomes (Cuadrado et al., 2008). Moreover, the presence of weak and single signals of GAA satellite sequence in chromosomes 1D, 2D, and 7D was also reported (Pedersen and Langridge, 1997; Komuro et al., 2013). On the other hand, fluorescence *in situ* hybridization (FISH) with repetitive sequences from BAC library of wheat (cv. Chinese Spring) revealed the presence of hybridization signals in D-genome and availability of sequences for *Ae. tauschii* karyotyping.

The main goal of this work was to examine the differences and similarities in chromosomes organization between the accessions of *Ae. tauschii* with a different origin in order to identify new sources of variation for breeding of Triticeae species especially resistance to fungal diseases. Therefore cytogenetic analyses were supported with molecular markers associated with genes determining resistance to leaf rust and powdery mildew.

## Materials and Methods

### Plant Material

The list of all accessions of *Ae. tauschii* used in this study with their origin is shown in **Table 1**. The materials were kindly provided by the United States Department of Agriculture, the Agricultural Research Service (Aberdeen, ID, United States), as well as were derived from the collection of the Institute of Plant Genetics, Polish Academy of Sciences (Poznań, Poland). For the molecular markers analysis triticale cv. Bogo, wheat cultivars Chinese Spring, Thatcher and NILs of wheat cv. Thatcher carrying *Lr22a, Lr22b* and *Lr39* genes, were used as control plants. Wheat cv. Thatcher and NILs of this cultivar were kindly provided by Dr. A. Serfling (Julius Kühn-Institut, Federal Research Centre for Cultivated Plants, Quedlinburg, Germany). All plants were grown in a greenhouse at the Institute of Plant Genetic of the Polish Academy of Sciences.

**Table 1:** List of *Aegilops tauschii* accessions used in the study.

		Accession		
No.	Genotype	number	Origin	GenBank^∗^
1	Tau-1	PI 486270	Turkey, Hakkari	USDA-ARS/USA
2	Tau-2	PI 486271	Turkey, Van	USDA-ARS/USA
3	Tau-3	PI 486272	Turkey, Van	USDA-ARS/USA
4	Tau-4	PI 486274	Turkey, Kars	USDA-ARS/USA
5	Tau-5	PI 486275	Turkey, Kars	USDA-ARS/USA
6	Tau-6	PI 486276	Turkey, Kars	USDA-ARS/USA
7	Tau-7	PI 486277	Turkey, Kars	USDA-ARS/USA
8	Tau-8	PI 499262	China, XinJiang	USDA-ARS/USA
9	Tau-9	PI 508261	China, XinJiang	USDA-ARS/USA
10	Tau-10	PI 508262	China, XinJiang	USDA-ARS/USA
11	Tau-11	PI 511362	Pakistan, Baluchistan	USDA-ARS/USA
12	Tau-12	PI 511363	Afghanistan, Faryab	USDA-ARS/USA
13	Tau-13	PI 511369	Iran, Mazandaran	USDA-ARS/USA
14	Tau-14	PI 554311	Turkey, Van	USDA-ARS/USA
15	Tau-15	D1	Unknown	IPG PAS/Poland
16	Tau-16	D2	Unknown	IPG PAS/Poland
17	Tau-17	D15	Unknown	IPG PAS/Poland
18	Tau-18	D17	Turkey	IPG PAS/Poland
19	Tau-19	D27	Unknown	IPG PAS/Poland
20	Tau-20	D51	Unknown	IPG PAS/Poland
21	Tau-21	D98	Unknown	IPG PAS/Poland

*^∗^USDA-ARS, United States Department of Agriculture, Agricultural Research Service, Aberdeen, ID, United States. IPG PAS, Institute of Plant Genetics, Polish Academy of Sciences, Poznań, Poland.*

### Chromosome Preparation and Labeling of Probes

Germination, metaphase accumulation, and fixation procedures were carried out according to Kwiatek et al. (2016a). The chromosome slides were obtained from root tips with squash method according to Hasterok et al. (2006) with minor modifications. Three root meristems were collected from five plants of each accession. Chromosome preparations were carried out from each of three root meristems. The following five repetitive sequences were used for FISH: pTa-86, pTa-465, pTa-535, pTa-k566, pTa-713, and microsatellite (GAA)_5_. Selected sequences were amplified from the clones derived from BAC library of wheat cv. Chinese Spring reported by Komuro et al. (2013) according to Kwiatek et al. (2016b). After amplification, all products were electrophoresed, stained, visualized and photographed to confirm their length before labeling. All five pTa sequences were labeled with nick translation kits according to the instructions provided by the producers. Probes pTa-535 and pTa-k566 were labeled using tetramethyl-5-dUTP-rhodamine (Roche), pTa-86 was labeled with digoxigenin-11-dUTP (Roche) and pTa-465 and pTa-713 were labeled by Atto647N (Jena BioScience). (GAA)_5_ oligoprobe was end-labeled with the enzyme terminal deoxynucleotidyl transferase (TdT) and had digoxigenin-modified nucleotide attached at the 5′ end (Sigma).

### Fluorescent *In Situ* Hybridization

FISH procedure was performed according to Kwiatek et al. (2016a) with minor modifications. Six probes [pTa-86, pTa-465, pTa-535, pTa-k566, pTa-713, and (GAA)_5_] were subjected to *in situ* hybridization on the same chromosome preparations. In the first FISH trial, pTa-86, pTa-535, pTa-713 probes were applied. The hybridization mixture (20 μl per slide) contained 90 ng of each probe in the presence of salmon sperm DNA, 50% formamide, 2× SSC, and 10% dextran sulfate, and was denatured at 70°C for 10 min and stored on ice for 5 min. Denaturation of slides and hybridization mixture was carried out at 70°C for 3 min and hybridization was performed at 37°C for 20 h. The probes labeled with digoxigenin were detected using anti-digoxigenin-fluorescein antibody (Roche). After acquisition of sites obtained for the first FISH trial, the slides were washed according to Heslop-Harrison (2000) with minor modifications (2 min× 15 min in 4× SSC Tween in 37°C and 1 min × 5 min in 2× SSC, at room temperature). Second FISH trial with pTa-k566, pTa-465 and (GAA)_5_ probes was made with the same conditions after reprobing. Each set of probes was tested on chromosomes of *T. aestivum* Chinese Spring, as a control. 10 metaphases per slide were analyzed and documented with an Olympus BX 61 automatic epifluorescence microscope supplied with Olympus XM10 CCD camera. Image processing was carried out using the Olympus Cell-F (version 3.1; Olympus Soft Imaging Solutions GmbH: Münster, Germany) imaging software and PaintShop ProX5 software (version 15.0.0.183; Corel Corporation, Ottawa, ON, Canada).

### PCR Amplification of Molecular Markers

Total genomic DNA was isolated from 2-weeks-old leaves of individual accessions of *Ae. tauschii*, triticale cv. Bogo, wheat cultivars Chinese Spring, Thatcher and NILs of this cultivar, using GeneMATRIX Plant and Funghi DNA Purification Kit (EURx, Ltd) according to the manufacturer instruction. Analyses were performed with 25 μl mixture according to the procedure supplied to the 2x PCR TaqNovaHS PCR Master Mix (Blirt). PCR reaction mix consist of Master mix (containing hot start polymerase), 10 μM of each forward and reverse primer, DNA template and PCR-grade water. PCR reactions were carried out according to producer protocol with appropriate annealing temperature (52–60°C) estimated for every molecular marker (**Table 2**). Amplification products were separate in agarose gel (Sigma), stained, visualized, and photographed according to Kwiatek et al. (2016b).

**Table 2:** Sequences and annealing temperatures for molecular markers associated with resistance genes used in the study.

Marker name (gene)	Primer sequence (5′–3′)	Annealing temperature (°C)	Reference
*Xcfd19* (*Lr32*)	TACGCAGGTTTGCTGCTT CT	59	Guyomarc’h et al., 2002
	GGAGTTCACAAGCATGGGTT		
*Xksu-D14* (*Lr32*)	CGCTTTTACCGAGATTGGTC	59	Boyko et al., 1999
	CCAAAGAGCATCCATGGTGT		
*Xgwm296* (*Lr22a, Lr39*)	AATTCAACCTACCAATCTCTG	52	Roder et al., 1998
	GCCTAATAAACTGAAAACGAG		
*Xgdm35* (*Lr22a, Lr39*)	CCTGCTCTGCCCTAGATACG	60	Pestsova et al., 2000
	ATGTGAATGTGATGCATGCA		
*Xcfd4* (*Lr32*)	TGCTCCGTCTCCGAGTAGAT	59	Guyomarc’h et al., 2002
	GGGAAGGAGAGATGGGAAAC		
*Xbarc135* (*Lr32*)	ATCGCCATCTCCTCTACCA	58	Song et al., 2005
	GCGAACCCATGTGCTAAGT		
*Xgwm539* (*Pm43 homolog*)	CTGCTCTAAGATTCATGCAACC	58	Roder et al., 1998
	GAGGCTTGTGCCCTCTGTAG		

## Results

### Distribution of Highly Repetitive DNA Sequences on Chromosomes

**FIGURE 1:**
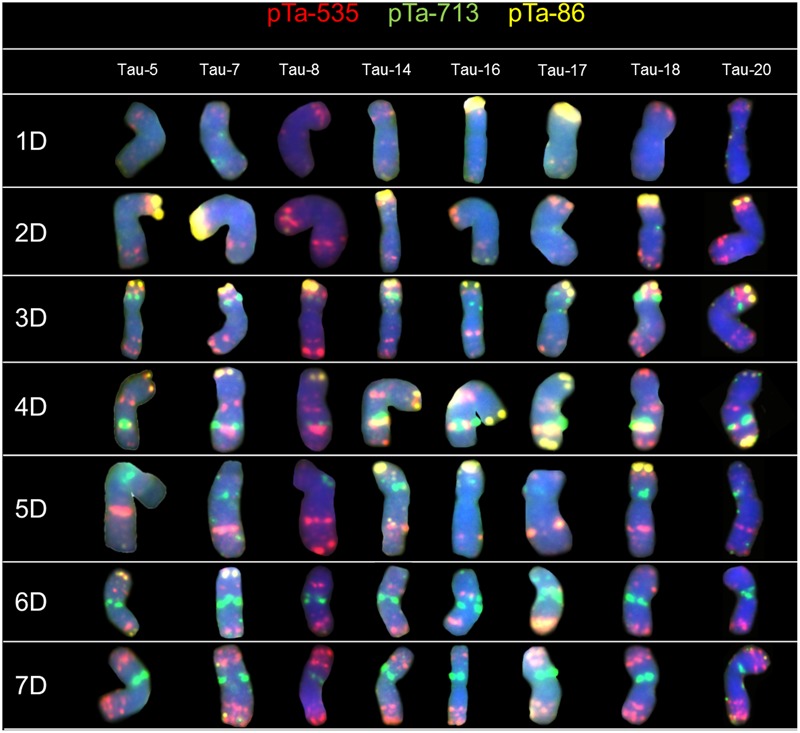
Karyograms of Tau-5, Tau-7, Tau-8, Tau-14, Tau-16, Tau-17, Tau-18, and Tau-20 after FISH with pTa-535 (red), pTa-86 (yellow), and pTa-713 (green) probes.

**FIGURE 2:**
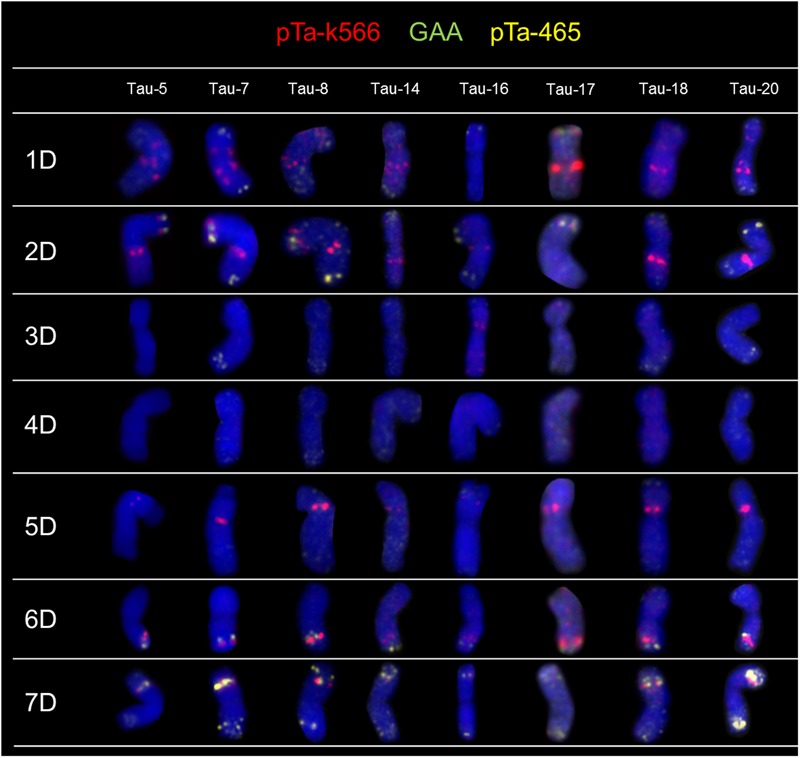
Karyograms of Tau-5, Tau-7, Tau-8, Tau-14, Tau-16, Tau-17, Tau-18, and Tau-20 after FISH with pTa-k566 (red), pTa-465 (yellow), and (GAA)_5_ (green) probes.

The FISH experiments were carried out on 21 accessions of *Ae. tauschii* in order to detect chromosome polymorphisms. To identify all chromosomes and genetic variation between analyzed accessions, two sets of probes were subjected to *in situ* hybridization: (1) pTa-86 + pTa-535 + pTa-713 (**Figure 1**) and (2) pTa-465 + pTa-k566 + (GAA)_5_ (**Figure 2**). The karyotypes were analyzed and obtained hybridization patterns were compared with that obtained for D-genome by Komuro et al. (2013). Most of the *Ae. tauschii* chromosomes showed similar hybridization patterns, but some differences were observed among accessions of different origin.

#### Sequence pTa-535

**FIGURE 3:**
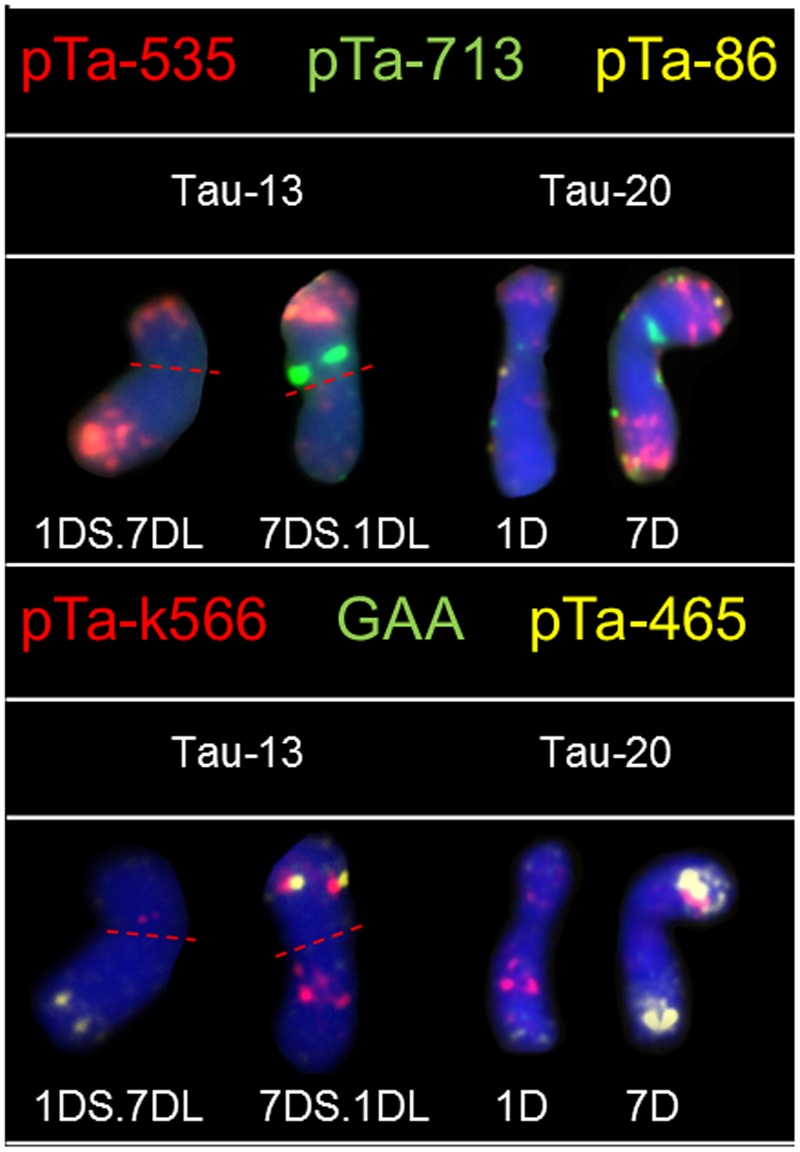
Karyograms of Tau-13 and Tau-20 after FISH with pTa-535 (red), pTa-86 (yellow), and pTa-713 (green) probes and reprobing with pTa-k566 (red), pTa-465 (yellow), and (GAA)_5_ (green) probes. Dotted lines indicate translocation breakpoints in centromeres.

Hybridization of *Ae. tauschii* chromosomes with pTa-535 revealed signals in all chromosomes, localized mainly in the telomeric regions. The hybridization pattern was similar in all accessions, however in chromosomes 3D (Tau-8, Tau-9, Tau-10) and 5D (Tau-4, Tau-17, Tau-18) the size of the sites located in the middle of the long arm was variable (**Figure 1**). On the basis of pTa-535 distribution in Tau-13 a reciprocal translocation between short arm of chromosome 1D and long arm of chromosome 7D was observed (**Figure 3**).

#### Sequence pTa-86

Patterns of pTa-86 probe were the most variable between analyzed accessions of *Ae. tauschii*. Intraspecific differences were found for chromosomes 1D, 2D, 4D, 5D, and 6D. The hybridization signal was observed in short arm of chromosome 1D only in Tau-15, Tau-16, and Tau-17 (**Figure 1**). For most of the accessions, signals observed in the terminal region of chromosome 2D were similar, however, these ones varied in Tau-1 more distinct signals (**Figure 1**); Tau-8, Tau-9, Tau-10 (less distinct signals), as well as Tau-16 and Tau-17 with the absence of hybridization signals (**Figure 1**). On chromosome 4D, signals of pTa-86 were detected mainly in the terminal part of short arm (4DS), though some accessions (Tau-17, Tau-20, Tau-21) possessed additional signals in the terminal region of long arm (4DL) (**Figure 1**). In two accessions Tau-3 and Tau-18 we did not reveal any signals for this probe in chromosome 4D (**Figure 1**). Moreover, the pTa-86 signals appeared in the terminal region of short arm of 5D chromosomes of Tau-2, Tau-3, Tau-4, Tau-6, Tau-14, Tau-15, Tau-16, Tau-18, and Tau-19 (**Figure 1**). What is more, this probe hybridized to the telomeric regions of chromosome 6D, concerning short arm of chromosomes of Tau-4 and Tau-7; long arm of Tau-2 and Tau-14 and both arms of Tau-5 (**Figure 1**).

#### Sequence pTa-713

Sites of pTa-713 probe were present in several chromosomes of *Ae. tauschii*. The most distinct signals were observed in the interstitial regions of chromosomes 3D, 4D and 5D, as well as in the pericentromeric regions of chromosomes 6D and 7D (**Figure 1**). Intraspecific variation in signal localization and intensity between accessions was not observed.

#### Sequence pTa-k566

Fluorescence *in situ* hybridization with pTa-k566 resulted in clear, distinct signals localized in the interstitial regions of chromosome 1D in both arms, chromosome 2D (both arms), chromosomes 5D and 6D (short and long arm, respectively), as well as in short arm of chromosome 7D (**Figure 2**). The hybridization pattern was similar in all accessions. Some differences were observed between analyzed accessions in the distribution of pTa-k566 probe in chromosome 1D. In accession Tau-16 one additional site of hybridization in the short arm of chromosome 3D was identified (**Figure 2**). The distribution pattern of pTa-k566 clone allows to confirm a reciprocal translocation between short arm of chromosome 1D and long arm of chromosome 7D in Tau-13 (**Figure 3**).

#### Sequence pTa-465

pTa-465 probe hybridized mainly to the distal regions of chromosomes 1D, 2D, 3D, 6D, and 7D (**Figure 2**). The most distinct signals were observed in terminal regions of both arms of chromosome 7D (**Figure 2**). The hybridization pattern was similar in all accessions. However, in Tau-14, Tau-15, Tau-18, Tau-19 and Tau-21, additional signals in the terminal region of the short arm of chromosome 6D, where this probe co-localized with pTa-k566 clone, were observed (**Figure 2**).

#### Sequence GAA

We have not revealed any GAA signals in chromosomes of all analyzed accessions of *Ae. tauschii* (**Figure 2**). All experiments with GAA motif as a probe, were also carried out on chromosomes of *T. aestivum* cv. Chinese Spring, as a positive control.

### Analysis of Molecular Markers Related to *Ae. tauschii* Leaf Rust Resistance Genes

Six molecular markers associated with leaf rust resistance genes [*Xcfd19* and *XksuD14* (*Lr32*, 1D); *Xgwm296* and *Xgdm35* (*Lr22a, Lr39*, 2D); *Xcfd4* and *Xbarc135* (3D)] and one marker linked with powdery mildew [*Xgwm539* (*Pm43* homolog, 2D)] resistance were used. The results showed the high polymorphism between all analyzed accessions of *Ae. tauschii*.

**FIGURE 4:**
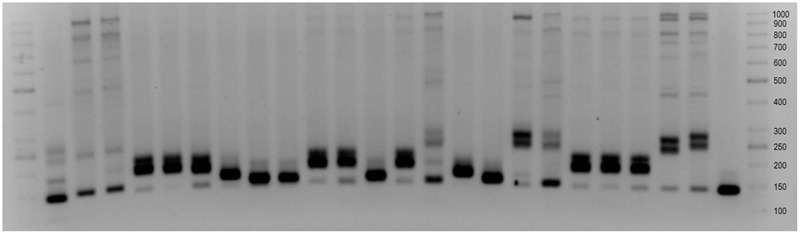
*Xgdm35* marker associated with *Lr39* gene (1) DNA Ladder 50 bp, (2–22) Tau-1-Tau-21, (23) Thatcher, (24) Thatcher-*Lr39*, (25) Bogo (26) DNA Ladder 50 bp.

Amplification of *XksuD14* marker resulted in 1.36 kb product indicating the presence of *Lr32* gene and was observed in all of *Ae. tauschii* accessions with expect of Tau-11, Tau-13, Tau-20 and Tau-21. What is more, 1.2 kb band was observed in Tau-1-Tau-9, Tau-14, Tau-15, Tau-17, and Tau-18. Similar banding patterns were obtained for Tau-10, Tau-11, Tau-12, Tau-13, Tau-20 and Tau-21, where a strong band of about 800 bp size was identified. This result was in accordance with similarities observed for the same accessions for *Xcfd19* marker, which is localized nearby *Lr21* gene. Results obtained for *Xgdm35* marker revealed that allele determining resistance (180 bp) was present in Tau-4, Tau-5, Tau-6, Tau-10, Tau-11, Tau-13, Tau-19, Tau-20, and Tau-21 (**Figure 4**). It is worth to mention, that the size of the bands differs between accessions of *Ae. tauschii* and NILs of *T. aestivum* cv. Thatcher with *Lr39*, however, one accession (Tau-18) had banding pattern similar to those obtained for Tc-*Lr39* (**Figure 4**). Presence of another resistant gene *Lr22*, localized in chromosome 2D was determined with *Xgwm296* marker. The banding pattern specific for resistance allele *Lr22a* was present in most of the analyzed accessions with expect of Tau-3 and Tau-16 which pattern was similar to those obtained for wheat cultivar Thatcher. In case of using *Xbarc135* and *Xcfd4* markers associated with *Lr32* gene, the high polymorphism of obtained banding patterns was revealed between all analyzed *Ae. tauschii* accessions. Amplification of *Xgwm539* marker resulted in a product of 150 bp size and was present in most of the analyzed accessions. What is more, three of them: Tau-15, Tau-16, and Tau-17 had banding pattern similar to wheat cultivars Chinese Spring and Thatcher.

It is worth pointing out that in case of all six markers linked to leaf rust resistance, Tau-10 had different organization of those loci in comparison with two other accessions of Chinese origin: Tau-8 and Tau-9. What is more, selected molecular markers revealed polymorphism within Turkish accessions. The most distinctive differences were observed in the comparison of banding patterns obtained for *Xbarc135, Xgdm35*, and *Xcfd19* markers. The differences and similarities in genome organization were in accordance with the origin of particular accessions of *Ae. tauschii*.

## Discussion

Komuro et al. (2013) reported that combination of pTa-86 + pTa-535 + pTa-713 probes allows to discriminate all D-genome chromosomes of wheat without ambiguity including their orientation. What is more, by overlaying pTa-465, pTa-k566 and GAA FISH signals, wheat D-genome chromosomes were highly decorated. The main goal of this study was to examine the differences and similarities in chromosomes organization among accessions of *Ae. tauschii* with a different origin, which is believed as a potential source of genes especially determining resistance to fungal diseases (i.e., leaf rust) that could be used in wheat and triticale breeding.

Results obtained for *Ae. tauschii* chromosomes revealed many similarities between analyzed accessions, however, some hybridization patterns were specific for accessions which become from cognate regions of the World. The most noticeable differences were observed for accessions from China which were characterized by presence of distinct signals of pTa-535 in the interstitial region of chromosome 3D, less intensity of pTa-86 signals in chromosome 2D, as well as lack of additional signals of pTa-86 in chromosomes 1D, 5D, or 6D (**Figure 1**). These results were in compliance with results of Wang et al. (2013) who reported that landraces of *Ae. tauschii* of Chinese region and other wheats of the Far Eastern origin appeared homogeneous and separate from landraces that originated in western Asia.

Chromosomes 1D and 2D of *Ae. tauschii* are of great importance because on this chromosomes many resistance genes have been mapped (Gill et al., 2008; Majka et al., 2016). In wheat, those chromosomes were found to be highly polymorphic, whereas in all wheat D-genome chromosomes most genetic diversity was localized in the terminal parts of chromosomes and was correlated with high recombination rates (Akhunov et al., 2010; Wang et al., 2013). It was assumed that wheat polymorphisms must have been introgressed into wheat from *Ae. tauschii* (Wang et al., 2013). The high content of genes is directly proportional to the low content of repetitive sequences along the entirety of chromosomes 1D and 2D what was in accordance with the scarcity of hybridization sites of pTa probes which are repetitive sequences. Especially terminal region of the short arm of chromosome 2D is characterized by high recombination rate. This region was reported to be exposed to chromosome rearrangements, though such places in the genome may constitute a hot spots (Luo et al., 2009, 2013; Majka et al., 2016). Tau-16 and Tau-17 are the only accessions that lacks signals of the pTa-86 signals in chromosome 2D (**Figure 1**). What is more, results obtained for the rest of *Ae. tauschii* chromosomes confirm the patterns of genetic diversity among D-genome chromosomes and arms of wheat where diversity is high in terminal region of the long arm of chromosome 4D, and both terminal regions of chromosome 6D. In the contrary, very low levels of diversity were reported for the whole chromosomes 3D and 5D, three-quarters of chromosome 4D, and terminal regions of 6D (Akhunov et al., 2010). Friebe et al. (1992) reported a large amount of polymorphic variation in C-bands size, as well as in the intra-chromosomal distribution pattern of these bands between different accessions of *Ae. tauschii*. It is known, that C-banded regions are composed of highly repetitive DNA sequences and the location of hybridization sites in this work are similar to the C-banding distribution in chromosomes of *Ae. tauschii* reported by Friebe et al. (1992). Moreover, it was shown, that *Ae. tauschii* TA 2462 (origin – Mazandaran province, Iran) carry a reciprocal translocation T1DS.7DL and T7DS.1DL basing on C-banding and hybridization patterns of pAs1 probe (Friebe et al., 1992). What is more, Hohmann and Lagudah (1993) using C-banding technique reported the same reciprocal interchange between chromosomes, with the breakpoints located in the centromeric region of *Ae*. *tauschii* accession AUS 18902 with Iranian origin. The same translocation was present in Tau-13 (this work; origin – Mazandaran province, Iran), what indicates a common origin of all accessions (**Figure 3**).

In this study *Ae. tauschii* chromosomes showed similar hybridization patterns to wheat D-genome chromosomes, however, some differences were also observed among both species. It is worth to mention, that combination of pTa-86 + pTa-535 + pTa-713 probes allows to discriminate all *Ae. tauschii* chromosomes and their orientation according to Komuro et al. (2013). Terminal parts of chromosomes 1DL, 5DS, and 6DS carried less distinct pTa-535 signals in comparison to wheat cv. Chinese Spring. The crucial differences concern the distribution and intensity of pTa-86 probe. The typical pattern for this repetitive sequence revealed signals in terminal regions of chromosomes (short arms) 2D, 3D, and 4D (**Figure 1**). What is more, in about 43% of analyzed accessions strong hybridization signals in short arm of chromosome 5D, were identified (**Figure 1**). In addition, no differences were observed for pTa-713 probe. The hybridization patterns of all probes established on Tau-13 chromosomes were similar to those observed in wheat Chinese Spring. This accession was collected in Mazandaran province in Iran, what is in relevance with the results obtained by Wang et al. (2013) who report that southwestern and southern Caspian accessions of *Ae. tauschii* appears to be the main source of the wheat D-genome.

pTa-86 clone is a homolog of pSc119.2 sequence which was derived from *S. cereale* (Bedbrook et al., 1980; Komuro et al., 2013). The typical hybridization pattern obtained for *Ae. tauschii* chromosomes revealed six strong hybridization signals in terminal parts of chromosomes 2D, 3D, and 4D (short arms) (**Figure 1**). That results were in coincidence with hybridization pattern of pSc119.2 obtained for *Ae. tauschii* accession MvGB605 by Molnar et al. (2014) and for accession Tau-20 by Kwiatek et al. (2013), where additional signals in terminal region of chromosome 4DL were also observed. However, both papers did not reveal any distinct signals of pSc119.2 in chromosomes 1D, 5D and 6D, what confirms that this hybridization pattern is specific only for some accessions with a different origin. Majka et al. (2017), hybridized gDNA of rye to chromosomes of *Ae. tauschii* (Tau-20) what reveals chromosome labeling and six terminal signals in chromosomes without 5S and 35SrDNA loci (1D and 5D). In this paper we also observed six distinct hybridization signals obtained for pTa-86 probe, what is consistent with GISH results and confirms homology of pTa-86 with pSc119.2 sequence derived from *S*. *cereale* (**Figure 1**).

High polymorphism between analyzed accessions and extensive allelic variation were revealed using molecular markers associated with resistance genes. In case of most of the markers localized in chromosomes 1D and 2D, the diversity of banding patterns was high. Additionally, results obtained for markers located in the distal part of the short arm of chromosome 3D also revealed the high diversity of obtained banding patterns. What is more, the differences in number and size of the bands were observed even between accessions with close origin. It should be mention that most of the analyzed molecular markers, thus resistance genes, were mapped to the distal parts of D-genome chromosomes (Somers et al., 2004; Liu et al., 2014). Only two of them *Xgwm296* and *Xgwm539* were mapped to the interstitial parts of chromosomes, but the polymorphism of obtained banding patterns was low. That confirms the high polymorphism of chromosomes 1D and 2D, as well as high recombination rates at the ends of D-genome chromosomes (Akhunov et al., 2010; Luo et al., 2013). What is more, Naghavi et al. (2009) using SSR markers reported that genetic variability in the genome of *Ae. tauschii* were higher than in D-genome of bread wheat. Therefore, this wild species might constitute a source of agronomically important genes that could be transferred into wheat germplasm (Naghavi et al., 2009). Singh et al. (2012) reported that only one of the known, major leaf rust resistance genes *Lr39* was declared to be associated with marker *Xgdm35*, through the conservative test. They declared that 180 bp product obtained for *Ae*. *tauschii* is directly related to the resistance. What is more, Wiśniewska et al. (2007) obtained 250 bp product for wheat cultivar Thatcher, whereas Sun et al. (2009) show that *Xgdm35* amplified polymorphic products (229–265 bp in range) in diverse wheat germplasm (239 bp in wheat cultivar Chinese Spring). This reports were in compliance with results obtained in this study and indicates that Tau-4, Tau-5, Tau-6, Tau-10, Tau-11, Tau-13, Tau-19, Tau-20, and Tau-21 can consist a source of leaf rust resistance, whereas Tau-18 has similar organization of *Xgdm35* locus in comparison to wheat cultivar Thatcher-*Lr39* (**Figure 4**).

## Conclusion

Obtained results imply, that there is a moderate or high level of polymorphism in the genome of *Ae. tauschii*. We showed the similarities and differences of repetitive sequences organization are determined by geographical origin. Therefore, selected accessions of *Ae. tauschii* might constitute an accessible source of variation for improvement of Triticeae species like wheat and triticale.

## Author Contributions

MM initiated and designed the study. MK and HW initiated and designed the project. MM and JM made the mitotic chromosome preparations followed by FISH analysis. MM performed the molecular marker analysis and analyzed the data. MM wrote the paper.

## Conflict of Interest Statement

The authors declare that the research was conducted in the absence of any commercial or financial relationships that could be construed as a potential conflict of interest.
